# Multilevel ecotoxicological responses of *Allium cepa* to atrazine and glyphosate applied individually and in combination

**DOI:** 10.1007/s10646-026-03156-y

**Published:** 2026-09-01

**Authors:** Karyne Marriel Moreira, Augusto César Santos Oliveira, Victor Ventura de Souza, Renata Alice Campos, José Marcello Salabert de Campos, Tatiana da Silva Souza

**Affiliations:** 1 Departamento de Biologia, Centro de Ciências Exatas, Naturais e da Saúde, Universidade Federal do Espírito Santo, Alegre, Espírito Santo Brasil; 2 Departamento de Biologia, Instituto de Ciências Biológicas, Universidade Federal de Juiz de Fora, Juiz de Fora, Minas Gerais Brasil

**Keywords:** Cytogenotoxicity, Flow cytometry, Mitochondrial activity, Membrane integrity, Phytotoxicity, Herbicide mixtures

## Abstract

Glyphosate and atrazine are among the most extensively applied herbicides worldwide and are often detected together in aquatic environments due to their concurrent agricultural use. This study investigated their individual and combined effects on *Allium cepa* at environmentally realistic concentrations: 62.5–1000 µg L⁻¹ for glyphosate and 0.25–4 µg L⁻¹ for atrazine. Five mixtures were also tested: M1 (62.5 + 0.25), M2 (125 + 0.5), M3 (250 + 1), M4 (500 + 2), and M5 (1000 + 4). Multiple endpoints were analyzed, including germination, root elongation, mitotic index, chromosomal and mitotic abnormalities, cell-cycle phase distribution, sub-G₁ fraction, fluorescence intensity, light-scattering parameters (FSC, SSC), membrane integrity (Evans Blue assay), and mitochondrial activity (TTC assay). Both herbicides and mixtures triggered cytotoxic and genotoxic responses, though most mixtures did not exceed the toxicity of the individual compounds, suggesting non-interactive or saturable mechanisms. Mixture M5, containing concentrations comparable to those reported in agricultural surface waters, produced stronger responses for some biomarkers. Flow-cytometric and mitochondrial endpoints were the most responsive following mitotic index and chromosomal aberrations. On the other hand, germination, root elongation, and membrane integrity showed limited sensitivity. Overall, the data indicate that even concentrations within current environmental guidelines can impair cellular function in *A. cepa*. This study provides a novel integrated assessment of glyphosate, atrazine, and their mixtures at environmentally relevant concentrations by combining classical *A. cepa* cytogenotoxicity assays with flow cytometry, membrane integrity, and mitochondrial activity biomarkers.

## Introduction

Brazil ranks among the world’s largest consumers of pesticides. Glyphosate (N-(phosphonomethyl)glycine) is the most widely used herbicide in the country, reflecting a global trend toward increasing dependence on this compound (Brovini et al. 2021; IBAMA, 2024). By inhibiting the synthesis of key enzymes essential for plant growth, glyphosate has proven highly effective in weed control. However, its intensive and recurrent use has resulted in the selection of resistant weed species, prompting farmers to adopt herbicide mixtures as an alternative management strategy (Bonfleur et al. 2015; Bordin et al. 2023).

Another widely applied herbicide in Brazil is atrazine (6-chloro-N2-ethyl-N4-isopropyl-1,3,5-triazine-2,4-diamine), which ranks third among the most commercialized agrochemicals in the country (Brovini et al. 2021; IBAMA, 2024). A member of the triazine family, atrazine inhibits Photosystem II (Teixeira et al. 2024) and is commonly used to control glyphosate-resistant weeds. Consequently, tank mixing of glyphosate and atrazine has become a routine agricultural practice adopted by many producers (Bonfleur et al. 2015; Bordin et al. 2023).

The widespread agricultural use of these herbicides is reflected in their frequent detection in aquatic environments (Hansen et al. 2019; Brovini et al. 2021, 2023; Soto-Verjel et al. 2023; Brown and Farenhorst, 2024; Cavassani et al. 2025). According to Brovini et al. (2023), glyphosate concentrations in surface Brazilian waters ranged from below the limit of quantification to 500 µg L⁻¹, with a mean of 56.7 µg L⁻¹ and a median of 50 µg L⁻¹, while particularly high values of 360–3700 µg L⁻¹ were reported in agricultural streams (Lima et al. 2023). Atrazine was detected in 10.9% of samples evaluated, reaching up to 3.3 µg L⁻¹ (mean = 0.3 µg L⁻¹), with the highest frequencies in the southern and southeastern regions, where maize and soybean production predominates and herbicide mixtures are widely applied (Brovini et al. 2023). Although these concentrations are environmentally realistic, they may approach or even exceed the maximum values permitted by Brazilian environmental legislation. For freshwater, Brazilian regulations establish maximum concentrations of 65 or 280 µg L⁻¹ for glyphosate, depending on the water quality category, and 2 µg L⁻¹ for atrazine (Brasil, 2005). For drinking water, the corresponding maximum permitted concentrations are 500 µg L⁻¹ for glyphosate and 2 µg L⁻¹ for atrazine (Brasil, 2021).

At the global scale, glyphosate has been reported at even higher concentrations, ranging from 1 to 25.2 mg L⁻¹ in agricultural runoff in Nigeria (Ayoola et al. 2023) and up to 105,000 µg L⁻¹ in Argentina (Sasal et al. 2017). Atrazine concentrations vary from 0.002 µg L⁻¹ in Spain (Borrull et al. 2021) and 0.23–3.9 µg L⁻¹ in Canada (Byer et al. 2011; Lahens et al. 2024) to 0.03–15.7 µg L⁻¹ (mean ≈ 0.2 µg L⁻¹) in the United States (Almberg et al. 2018). Together, these data highlight the potential risk for non-target organisms, even at concentrations close to or below regulatory thresholds.

The cytogenotoxic effects of glyphosate and atrazine are well documented, as both compounds induce DNA strand breaks, chromosomal aberrations, and mitotic disturbances in various organisms (Rank et al. 1993; Ventura et al. 2008; Chaufan et al. 2014; Woźniak et al. 2018; Rodrigues et al. 2019; Souza et al. 2023; Deng et al. 2024; Martins et al. 2025; Miranda et al. 2026). Nevertheless, studies addressing their combined effects remain limited. Roustan et al. (2014) showed that a mixture containing 5 mg L⁻¹ of each herbicide induced oxidative stress and micronucleus formation in mammalian ovary cells. Similarly, Bordin et al. (2023) observed that mixtures containing 2 µg L⁻¹ atrazine + 65 µg L⁻¹ glyphosate and 160 µg L⁻¹ glyphosate + 25 µg L⁻¹ atrazine increased chromosomal aberrations, micronucleus frequency, and reduced mitotic activity in *Allium cepa*.

The cytogenotoxic effects of such mixtures may vary according to the concentrations, proportions, and biological endpoints evaluated (Finkler et al. 2022; Souza et al. 2024). Hence, evaluating multiple mixture scenarios is essential to replicate realistic contamination gradients, from environmentally permissible to critical exposure levels. Accordingly, the present study investigated the effects of five glyphosate + atrazine mixtures on *A. cepa*, a well-established bioindicator of water quality (Matos et al. 2017; Vergilio et al. 2021; Romeiro dos Santos et al. 2023; Marques et al. 2025).

To achieve this objective, the effects of the individual and combined herbicides were evaluated using endpoints spanning multiple levels of biological organization: (a) germination rate and root growth, indicative of acute toxicity and developmental inhibition (US EPA, 1996); (b) mitotic index, reflecting alterations in cell division; (c) frequencies of chromosomal abnormalities, as indicators of genotoxicity (Leme and Marin-Morales, 2009; Camilo-Cotrim et al. 2022); (d) cell-cycle phase distribution (G1, S, G2) and cell death assessed by flow cytometry (Andrade-Vieira et al. 2012; Marques et al. 2025); and (e) cell viability inferred from membrane integrity and mitochondrial activity (Ghosh et al. 2015; Chakrabarti and Mukherjee, 2021).

By integrating traditional *A. cepa* bioassays with sensitive endpoints derived from flow cytometry, membrane integrity, and mitochondrial activity assays, this study extends the evaluation of glyphosate and atrazine mixtures beyond conventional cytogenetic analysis, providing more comprehensive information on the impact of these herbicides on non-target organisms.

## Material and methods

### Experimental design

The study was designed to compare the biological responses induced by individual herbicide exposures with those observed under combined application, allowing mixture effects to be interpreted as greater than, lower than, or comparable to those produced by the single compounds, in accordance with the criteria proposed by Finkler et al. (2022). To this end, five experimental groups were defined, each consisting of one glyphosate treatment, one atrazine treatment, and the respective mixture of both herbicides (Table 1).

**Table 1:** Experimental grouping and corresponding glyphosate and atrazine concentrations used for individual and combined treatments

Experimental group	Glyphosate (μg L^−1^)	Atrazine (μg L^−1^)	Corresponding mixture
Group 1	62.5	0.25	M1
Group 2	125	0.5	M2
Group 3	250	1	M3
Group 4	500	2	M4
Group 5	1000	4	M5

### Herbicides: concentrations and tested mixtures

Glyphosate (Pestanal®, purity 99%, CAS no. 1071-83-6) and atrazine (Pestanal®, purity 99%, CAS no. 1912-24-9) were purchased from Sigma-Aldrich (São Paulo, Brazil). The tested concentrations were selected according to the maximum permissible levels in freshwater established by CONAMA Resolution No. 357/2005 (Brasil, 2005) and in drinking water as defined by Ministry of Health Ordinance GM/MS No. 888/2021 (Brasil, 2021). In addition, concentrations above the legal limits were included to simulate critical contamination scenarios.

Glyphosate was tested at 62.5, 125, 250, 500, and 1000 μg L⁻¹, while atrazine was tested at 0.25, 0.5, 1, 2, and 4 μg L⁻¹ (Table 1). The mixtures were prepared by combining each glyphosate concentration with the corresponding atrazine concentration at a 1:1 (v:v) ratio, following Finkler et al. (2022).

### *A. cepa* bioassay

Seeds of *A. cepa* (Isla® Sementes, Porto Alegre, Brazil), variety Baia Periform, from the same batch, with a germination rate of 98% and untreated with pesticides, were used as the biological model for all assays. Twenty seeds were placed in each Petri dish (150 × 15 mm) lined with filter paper moistened with 3 mL of the respective test solution or with distilled water for the negative control. In addition, endpoint-specific positive controls were included to verify the responsiveness and proper functioning of the experimental system. Colchicine (0.025%) was used for germination, root growth, mitotic index, chromosomal abnormality, and flow cytometry analyses (Souza et al. 2021; Barman and Ray, 2023); hydrogen peroxide 1% was employed in the TTC assay; and zinc sulfate heptahydrate (6 mg L⁻¹) was used in the Evans Blue assay (Camilo-Cotrim et al. 2026). Because the primary objective of this study was to compare the responses induced by the herbicides and their mixtures, positive-control data were not included in the main text. However, comparisons between negative and positive controls are provided in the Supplementary Material (S1).

The dishes were kept in complete darkness at 24 ± 2 °C for 96 h, until primary roots reached approximately 1.5–2 cm in length, indicating suitable development for subsequent analyses (Anacleto et al. 2017; Felisbino et al. 2018). A separate bioassay was conducted for each methodology. All bioassays were conducted in triplicate under identical conditions to ensure methodological consistency and reproducibility.

### Germination and root length analysis

Seeds were classified as germinated once the reactivation of embryonic growth was confirmed by the protrusion of the primary root to a minimum length of 5 mm. This threshold was adopted in accordance with the EPA OPPTS 850.4200 guideline for the Seed Germination/Root Elongation Toxicity Test (US EPA, 1996). The germination rate (G) was subsequently obtained by calculating the proportion of germinated seeds relative to the total number of seeds distributed in each Petri dish, using the following equation:

$$G\left( \% \right)=\frac{{Number}\,{of}\,{seeds}\,{that}\,{germinated}\,}{{Total}\,{number}\,{of}\,{seeds}\,}x100$$

Root length (RL) of the main root was measured using a digital caliper (precision 0.01 mm).

### Cytogenotoxic analysis

Root tips were fixed in freshly prepared ethanol-acetic acid (3:1, v/v) solution. The samples were rinsed three times in distilled water, hydrolyzed in 1 N HCl for 8 min at 60 °C, and rinsed again. Subsequently, the roots were subjected to the Feulgen reaction by immersion in Schiff’s reagent for 2 h in the dark. Meristematic tissues were then sectioned, stained with 1% aceto-carmine, and squashed between slide and coverslip (Leme and Marin-Morales, 2008).

For permanent slide preparation, coverslips were detached in liquid nitrogen, and slides were sealed with Canada balsam. Two slides were prepared per Petri dish, totaling six slides (one root per slide) per treatment. A total of 1000 cells were analyzed per slide, resulting in 6000 cells per treatment (Bernardes et al. 2019; Banerjee et al. 2021). Observations were made under a light microscope (Leica DM500) at 400× magnification, equipped with a Leica ICC50 HD digital camera (Leica Microsystems).

The mitotic index (MI), chromosomal abnormalities (CA), and the frequency of individual alterations (IA) were calculated according to the following equations:

$${MI}\left( \% \right)=\frac{{Number}\,{of}\,{cells}\,{in}\,{division}\,}{{Total}\,{number}\,{of}\,{cells}\,{observed}}\,x100$$

$${AC}\left( \% \right)=\frac{{Number}\,{of}\,{cells}\,{with}\,{chromosomal}\,{abnormalities}\,}{{Total}\,{number}\,{of}\,{cells}\,{observed}}\,x100$$

$${IA}\left( \% \right)=\frac{{Number}\,{of}\,{cells}\,{with}\,a\,{specific}\,{chromosomal}\,{abnormalitie}}{{Total}\,{number}\,{of}\,{cells}\,{observed}}\,x100$$

Representative images of cell-cycle phases and the observed abnormalities were recorded for documentation.

### Flow cytometry analysis

Root apices not previously fixed were randomly sampled from the Petri dishes for nuclear isolation. Nuclear suspensions were obtained following the protocol described by Galbraith et al. (1983), with slight adaptations. Briefly, meristematic regions were carefully excised and mechanically dissociated using a scalpel in 600 µL of WPB buffer maintained on crushed ice to ensure nuclear integrity. The resulting homogenate was passed through a 40 µm nylon mesh to remove cellular debris. Subsequently, 50 µL of propidium iodide solution (1 mg L⁻¹) was added for DNA staining. For each treatment, one nuclear suspension was prepared from ten roots collected from each Petri dish. Since the bioassays were conducted in triplicate, three independent nuclear suspensions (biological replicates) were analyzed per treatment. Fluorescence signals were acquired using a CytoFLEX flow cytometer (Beckman Coulter), and 10,000 nuclei (events) were recorded per sample. Cell cycle phase distribution was determined from DNA content histograms, allowing discrimination of G₁ (2C), S, and G₂ (4C) nuclear populations. Events exhibiting DNA content below the 2C level were also recorded as indicative of cell death. Additional cytometric variables included the absolute fluorescence intensity of G₁-phase nuclei (IFa), along with forward scatter (FSC) and side scatter (SSC), used as proxies for nuclear size and internal complexity, respectively. The coefficient of variation (CV) of the G₁ peak was likewise calculated (Souza et al. 2023; Marques et al. 2025).

### Membrane integrity

Plasma membrane integrity was evaluated by Evans blue uptake according to the method of Ghosh et al. (2015), with slight modifications. Briefly, *A. cepa* root were incubated in a 0.25% (w/v) Evans blue solution for 15 min, followed by rinsing in distilled water for 30 min to eliminate excess dye. Subsequently, ten root fragments (approximately 1 cm in length) were transferred to N,N-dimethylformamide and maintained at room temperature for 1 h to promote dye extraction. The samples were then centrifuged at 3000 rpm for 3 min, allowing the release of dye retained by structurally compromised cells. Absorbance of the supernatant was recorded at 600 nm using a spectrophotometer (IL-226-nm Kazuaki). Since Evans blue is excluded by intact plasma membranes, increased absorbance values were interpreted as indicative of enhanced membrane permeability and cellular damage (Ghosh et al. 2015; Romeiro dos Santos et al. 2023).

### Mitochondrial activity

Mitochondrial activity was evaluated using the 2,3,5-triphenyltetrazolium chloride (TTC) reduction assay. Ten root tips in triplicate were immersed in a 0.5% TTC solution, the reagent was dissolved in 0.05 M phosphate buffer (pH 7) and incubated in the dark at 35 °C for 15 min. After the incubation period, the roots were washed with distilled water and the triphenylformazane red complex formed in viable tissues was extracted with 95% ethanol for 21 h at a temperature of 21 ± 1 °C under slight agitation. The optical density of the extract was measured espectrophotometrically at 490 nm. TTC is a water-soluble, colorless salt that is reduced to red formazan by mitochondrial dehydrogenases in metabolically active cells. Therefore, lower absorbance values in treated groups indicate reduced mitochondrial activity and cellular viability (Ghosh et al. 2015; Bibi et al. 2019; Chakrabarti and Mukherjee, 2021).

### Statistical analysis

Prior to inferential testing, data distribution for each endpoint was examined using the Shapiro-Wilk test to determine compliance with normality assumptions. Depending on the outcome, parametric or non-parametric approaches were adopted. Normally distributed data were analyzed by one-way analysis of variance (ANOVA), followed by Tukey’s multiple comparisons test (p < 0.05), whereas non-normally distributed data were evaluated using the Kruskal-Wallis test with Dunn’s post hoc comparisons (p < 0.05). These analyses were used to determine whether exposure to the individual herbicides or their mixtures resulted in responses significantly different from the control, as well as to compare the effects of each mixture with those induced by glyphosate or atrazine applied separately. All statistical analyses and graphical representations were generated using GraphPad Prism version 9.4 (GraphPad Software, San Diego, CA, USA).

## Results

### Germination and root growth analysis

**Fig. 1:**
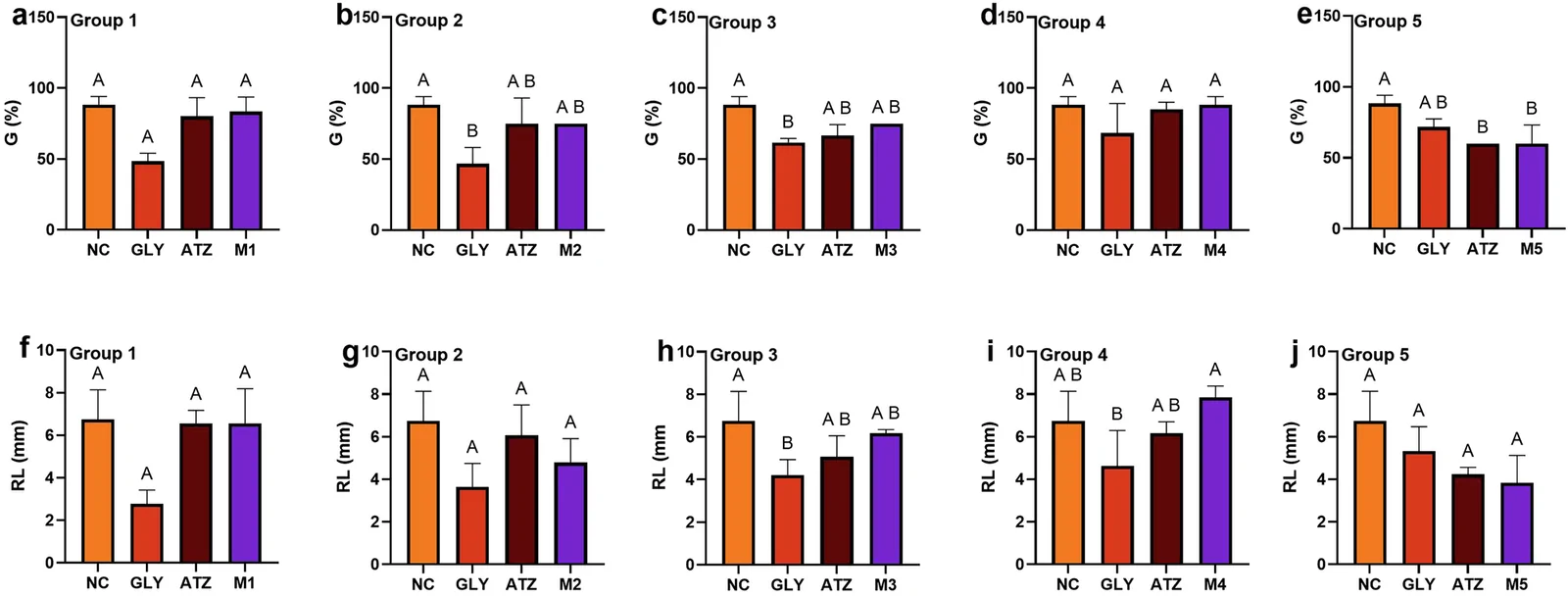
Phytotoxic responses of *A. cepa* following exposure to glyphosate (Gly), atrazine (ATZ), and their mixtures (M1–M5). **a**-**e** Seed germination (% G). **f**-**j** Root length (RL). Results are shown for the five experimental groups. Bars represent mean ± standard deviation. Treatments sharing the same letter do not differ significantly within each group (p < 0.05). NC negative control

The germination percentage and root length were used as indicators of the phytotoxic effects of glyphosate, atrazine, and their mixtures on *A. cepa* (Fig. 1).

When tested individually, glyphosate at 125 µg L⁻¹ (group 2) and 250 µg L⁻¹ (group 3), as well as atrazine at 4 µg L⁻¹ (group 5), significantly reduced the percentage of germinated seeds compared with the control. Among the mixtures, only M5 (group 5) inhibited seed germination relative to the control (Fig. 1a–e).

Regarding root growth, glyphosate at 250 µg L⁻¹ (group 3) and 500 µg L⁻¹ (group 4) caused a significant decrease in root length compared with the control, whereas all other treatments, both the individual herbicides and their mixtures, did not induce significant changes (Fig. 1f–j).

### Cytogenotoxic analysis

**Fig. 2:**
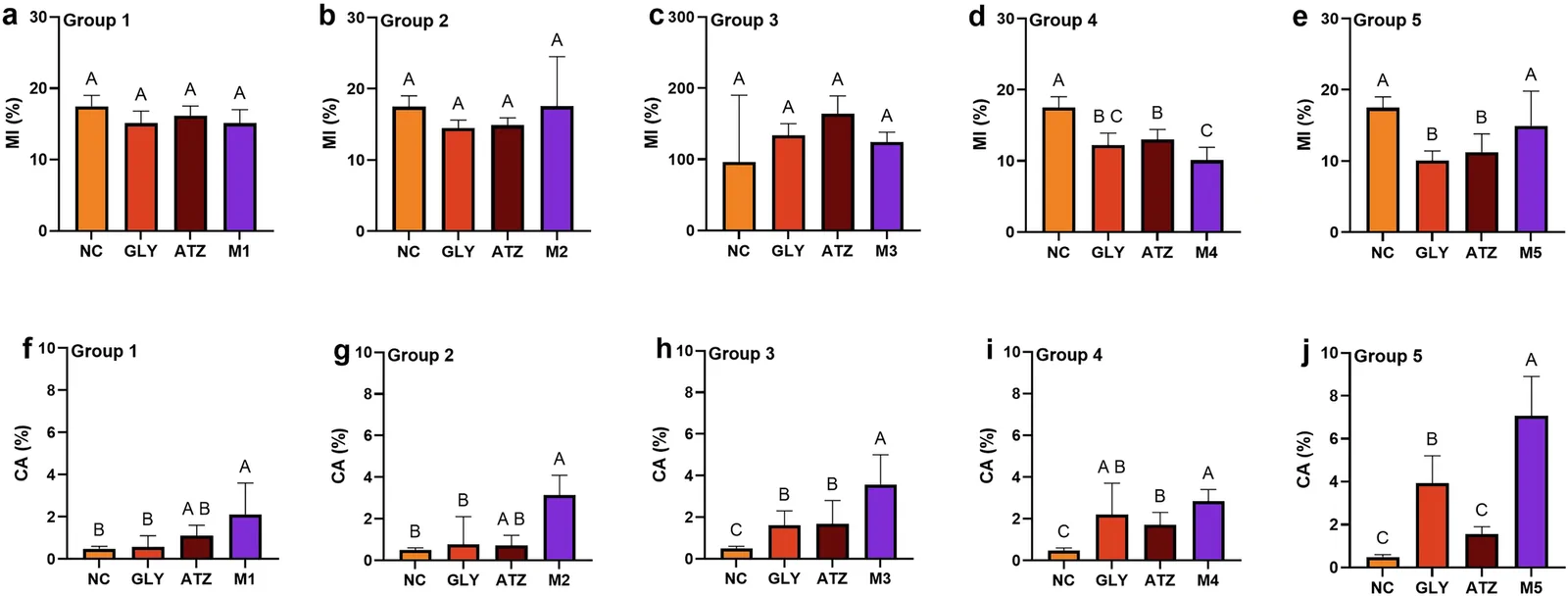
Cytogenotoxicity analysis on *A. cepa* following exposure to glyphosate (Gly), atrazine (ATZ), and their mixtures (M1–M5). **a**-**e** Mitotic index (MI). **f**-**j** Chromosomal abnormalities (CA). Results are shown for the five experimental groups. Bars represent mean ± standard deviation. Treatments sharing the same letter do not differ significantly within each group (p < 0.05). NC negative control

Figure 2 shows the effects of the treatments on the mitotic index and the frequency of mitotic and chromosomal alterations in *A. cepa*.

No significant changes in the mitotic index were observed at the lowest concentrations tested (groups 1 and 2). In contrast, intermediate and high concentrations of glyphosate and atrazine significantly reduced mitotic activity (Fig. 2a–e). Among the mixtures, only M3 and M4 decreased the mitotic index, whereas M5 did not affect this parameter despite being the most concentrated mixture (Fig. 2c–e).

Chromosomal abnormalities increased predominantly at intermediate and high concentrations of both herbicides, whereas all mixtures significantly increased the frequency of alterations relative to the control (Fig. 2f–j). The genotoxic effects of the mixtures generally increased with concentration and were comparable to or greater than those induced by the individual herbicides. M5 produced the highest frequency of abnormalities among all treatments (Fig. 2f–j).

**Fig. 3:**
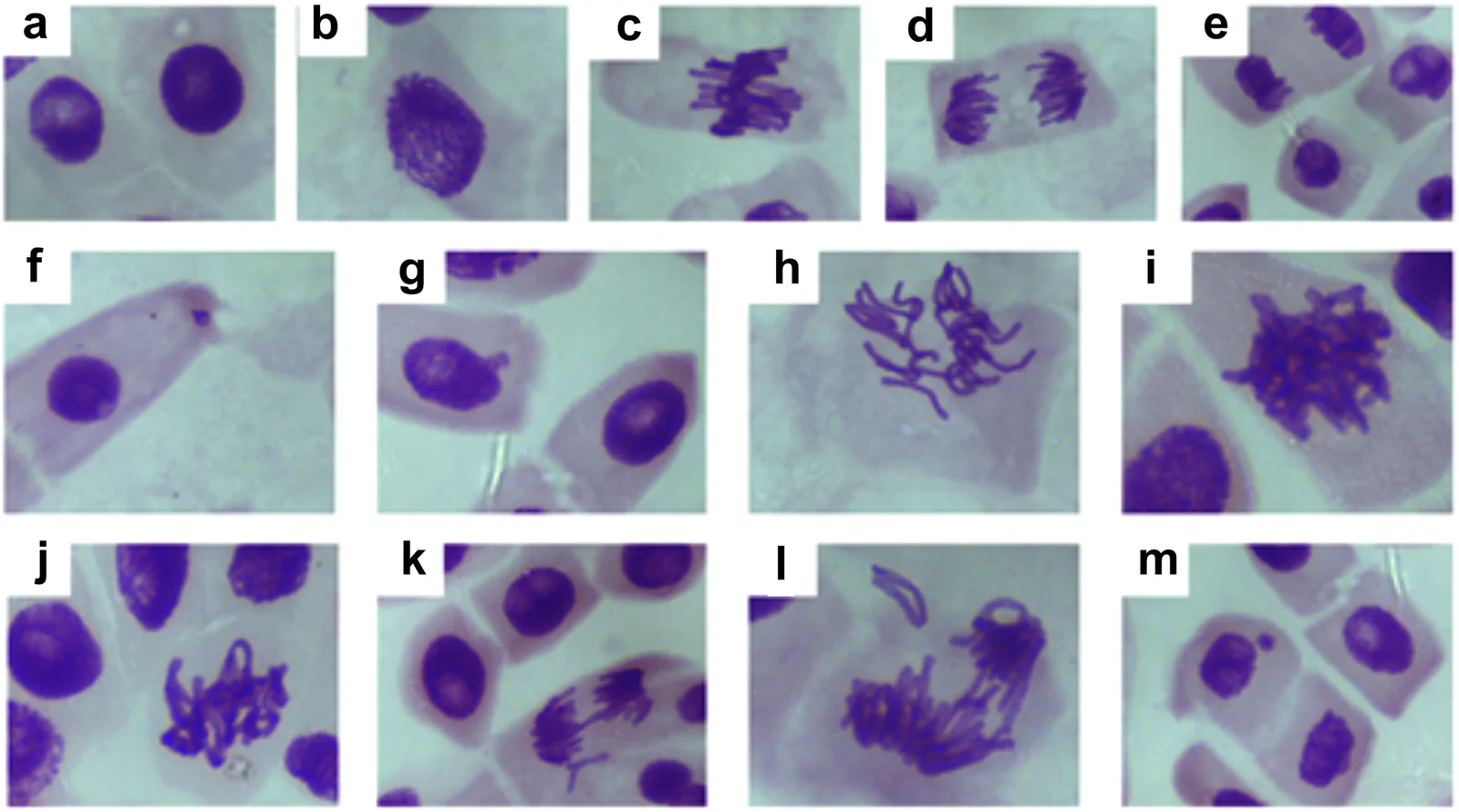
Normal cell-cycle phases of *A. cepa* **a**–**e** and major chromosomal abnormalities observed after exposure to glyphosate, atrazine, and their mixtures **f**–**m**. **a** Interphase. **b** Prophase. **c** Metaphase. **d** Anaphase. **e** Telophase. **f** Interphase cell with micronucleus. **g** Interphase cell with nuclear bud. **h** C-metaphase. **i**–**j** Chromosomal adhesion. **k**-**l** Anaphase bridge with chromosomal loss. **m** Cell with micronucleus

The relative frequency of the observed chromosomal alteration types is presented in Table S2. Overall, micronuclei and chromosomal breaks were the most frequent abnormalities contributing to the genotoxic effects of the treatments. Representative images of main alterations observed are shown in Fig. 3.

### Flow cytometry analysis

**Fig. 4:**
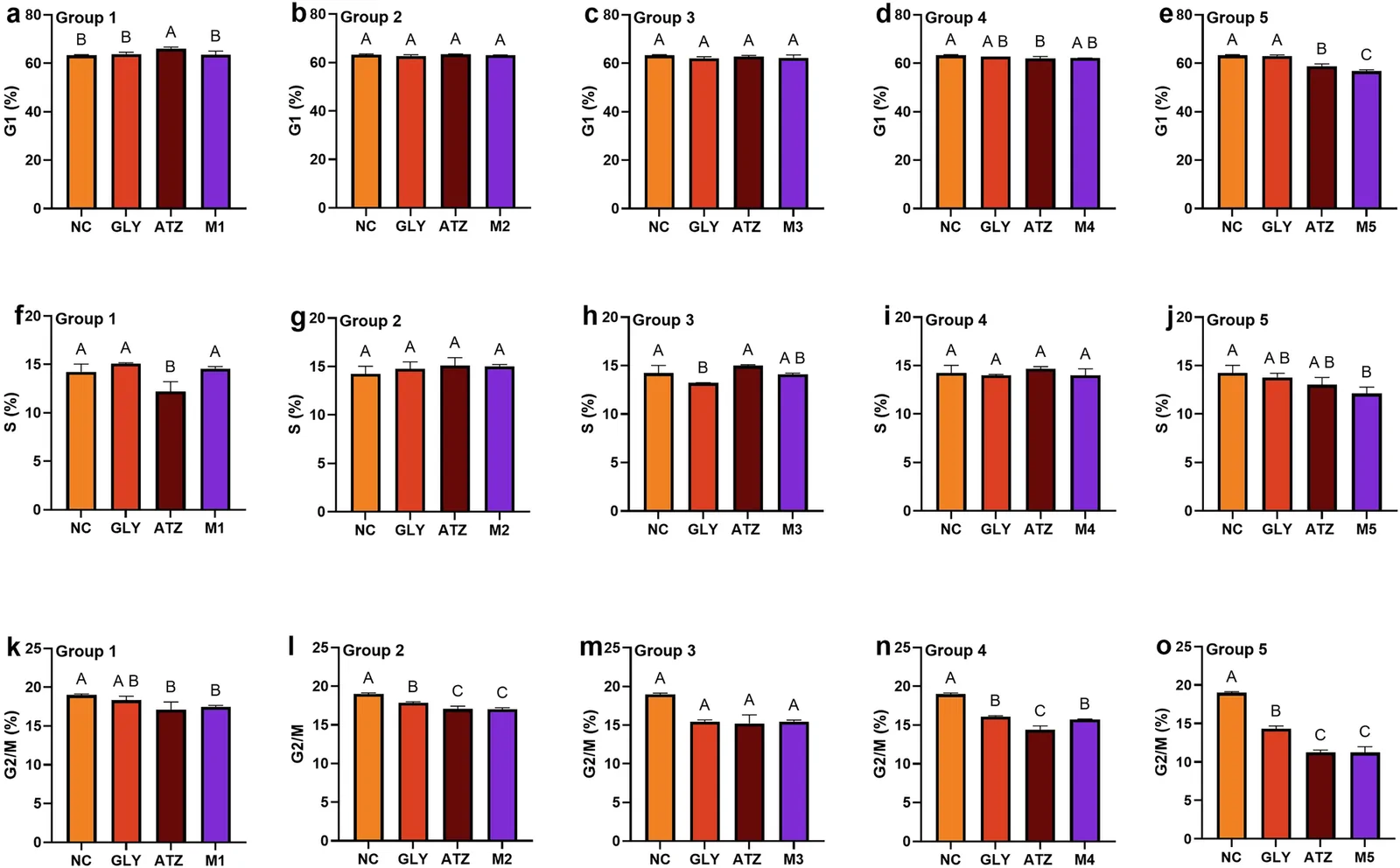
Cell cycle analysis by flow cytometry following exposure of *A. cepa* roots meristems to glyphosate (Gly), atrazine (ATZ), and their mixtures (M1–M5). **a**-**e** Frequency of nuclei in G_1_ phase. **f**-**j** Frequency of nuclei in S phase. **k**-**o** Frequency of nuclei in G_2_/M phase. Results are shown for the five experimental groups. Bars represent mean ± standard deviation. Within each experimental group, treatments marked with the same letter do not differ significantly (p < 0.05). NC negative control

The effects of the herbicides and their mixtures on the distribution of nuclei across the G₁, S, and G₂/M phases of the cell cycle are shown in Fig. 4. Significant changes in the frequency of G₁ nuclei were observed exclusively for atrazine, which increased the G₁ fraction at 0.25 µg L⁻¹ and reduced it at 2 and 4 µg L⁻¹, and for mixture M5, which produced the strongest decrease in G₁ frequency among all treatments (Fig. 4a–e).

Regarding the S phase, only atrazine (0.25 µg L⁻¹) in group 1 (Fig. 4f) and glyphosate (250 µg L⁻¹) in group 3 (Fig. 4h) caused a significant reduction in the proportion of nuclei undergoing DNA synthesis compared with the control. Among the mixtures, only M5 decreased the S-phase frequency; however, this effect did not differ significantly from those produced by the individual herbicides (Fig. 4j).

For the G₂/M phase, significant reductions in the proportion of nuclei were observed for several atrazine and glyphosate treatments, as well as for most mixtures. In general, mixture-induced responses were comparable in magnitude to those observed for the individual herbicides (Fig. 4k–o).

**Fig. 5:**
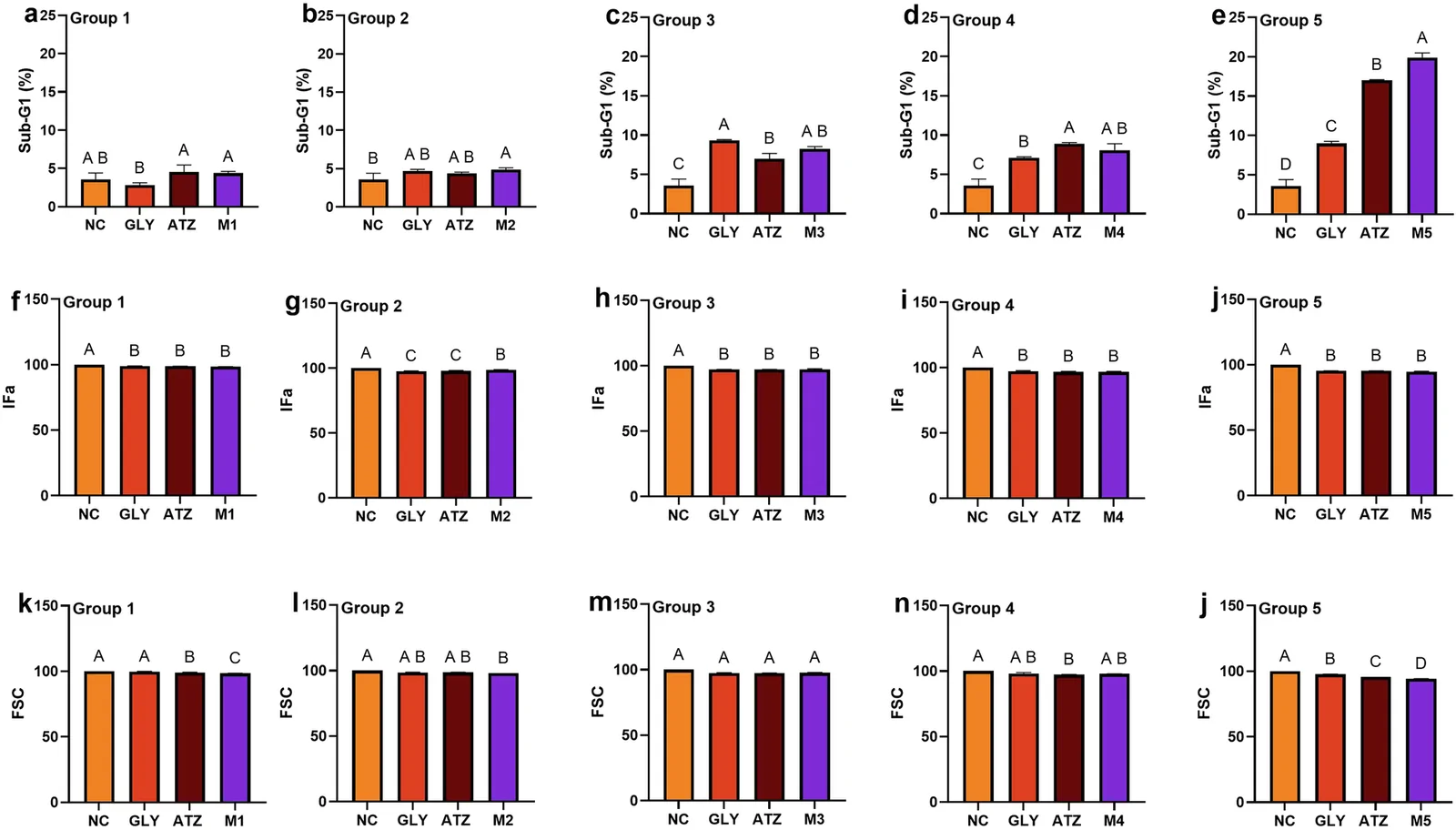
Flow-cytometric assessment of cell death in *A. cepa* root meristems following exposure to glyphosate (Gly), atrazine (ATZ), and their mixtures (M1-M5). **a**-**e** Frequency of sub-G_1_ particles. **f**-**j** Fluorescence intensity (IFa). **k**-**o** Nuclear diameter (FSC). Results are shown for the five experimental groups. Bars represent mean ± standard deviation. Within each experimental group, treatments marked with the same letter do not differ significantly (p < 0.05). NC negative control

**Fig. 6:**
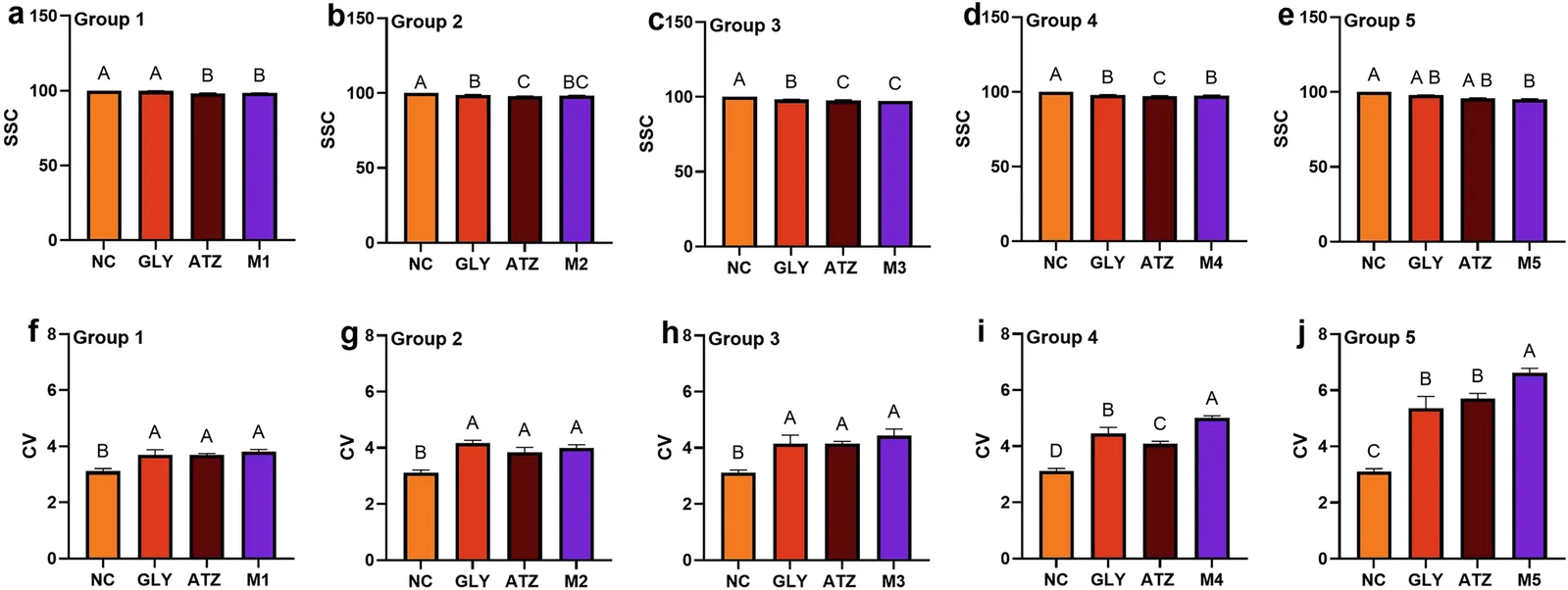
Flow-cytometric assessment of nuclear complexity (SSC) and G1 coefficient of variation (CV) in *A. cepa* root meristems following exposure to glyphosate (Gly), atrazine (ATZ), and their mixtures (M1–M5). **a**–**e** show nuclear complexity (SSC), and **f**–**j** present the coefficient of variation of the G1 peak (CV). Data are presented for the five experimental groups. Bars represent mean ± standard deviation. Within each experimental group, treatments sharing the same letter do not differ significantly (p < 0.05). NC negative control

In addition to cell cycle distribution, other flow cytometric parameters related to cell death were also analyzed, including the sub-G₁ fraction, fluorescence intensity in G₁, and changes in forward (FSC) and side (SSC) scatter signals (Figs. 5, 6).

Significant increases in the sub-G₁ fraction were observed mainly at intermediate and high concentrations of glyphosate, atrazine, and their mixtures, indicating increased cell death (Fig. 5b–e). M2, M3 and M4 produced responses similar to those observed for individual herbicides (Fig. 5b-d). M5 induced a greater increase in sub-G₁ nuclei than either herbicide alone (Fig. 5e).

All treatments decreased the fluorescence intensity of G₁ nuclei compared with the control (Fig. 5f-j). The responses to M1, M3, M4, and M5 were similar to those observed for glyphosate and atrazine (Fig. 5f, h–j), whereas M2 produced a significantly different pattern relative to both herbicides (Fig. 5g).

Both FSC and SSC values were reduced following exposure to glyphosate, atrazine, and their mixtures (Figs. 5k–o and 6a–e). Significant decreases in FSC were observed for selected herbicide concentrations and mixtures, whereas SSC was more sensitive, showing significant reductions for most herbicide treatments and all mixtures. Overall, mixture-induced responses were generally comparable to those observed for the individual herbicides, although M1 and M5 produced the most pronounced decreases in FSC.

All treatments increased the G₁ coefficient of variation (CV) compared with the control (Fig. 6f-j). M1, M2, and M3 induced changes equivalent to those produced by glyphosate and atrazine (Fig. 6f-h), whereas M4 and M5 caused more pronounced alteration (Fig. 6i-j).

### Membrane integrity

**Fig. 7:**
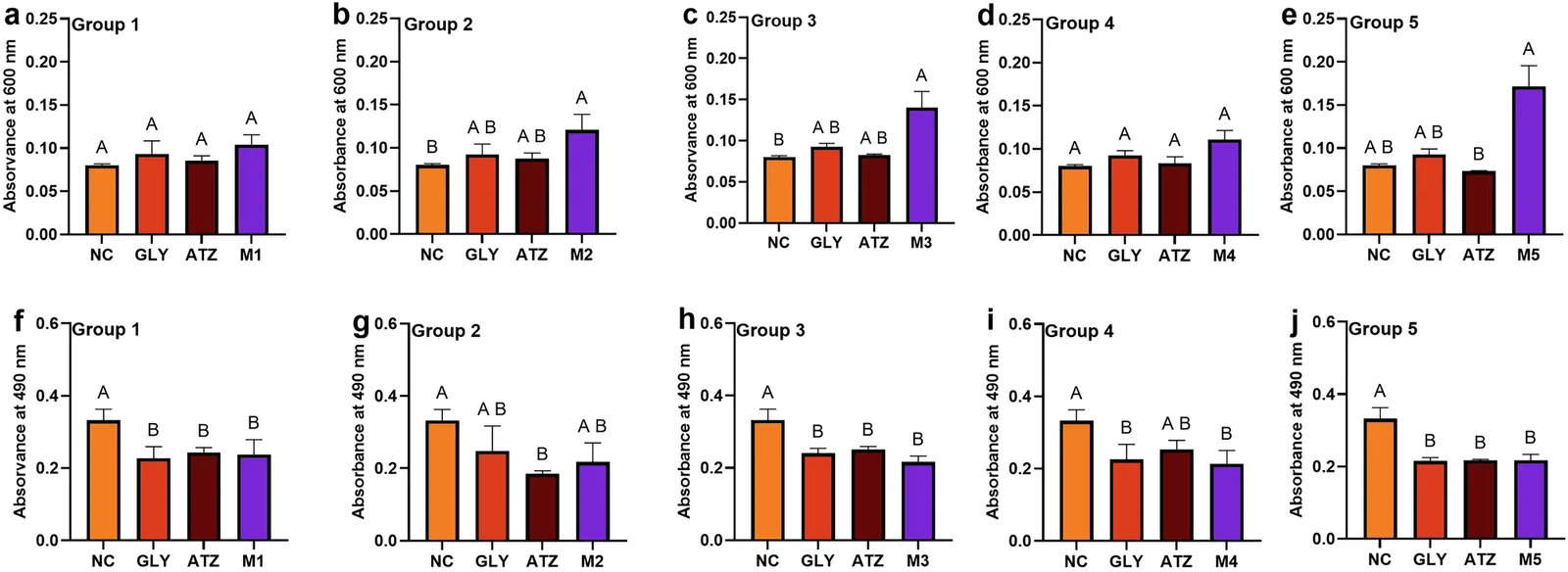
Membrane integrity and mitochondrial activity in *A. cepa* root cells exposed to glyphosate (Gy), atrazine (ATZ) and their mixtures (M1–M5). **a**–**e** Membrane integrity assessed by the Evans Blue assay (absorbance at 600 nm) for Groups 1–5. **f**–**j** Mitochondrial activity measured by the TTC reduction assay (absorbance at 490 nm) for the corresponding groups. Bars represent mean ± standard deviation. Treatments sharing the same letter do not differ significantly (p < 0.05). NC = negative control

Exposure to the individual herbicides did not significantly affect Evans Blue uptake. Among the mixtures, significant increases were observed only for M2 and M3, whereas M5 exhibited the highest absorbance values without reaching statistical significance (Fig. 7a–e).

### Mitochondrial activity

Figure 7f-j shows the effects of the treatments on mitochondrial activity in *A. cepa*. Root cells exposed to herbicides and mixtures in groups 1, 3, and 5 were unable to reduce 2,3,5-triphenyltetrazolium chloride to triphenyl formazan, indicating impairment of the mitochondrial respiratory chain, as evidenced by a significant decrease in optical density relative to the control (Fig. 7f, h, j).

In group 2, only atrazine (0.5 µg L⁻¹) caused a significant reduction in optical density compared with the control (Fig. 7g), whereas in group 4, this reduction was observed for glyphosate (500 µg L⁻¹) and mixture M4 (Fig. 7i). Across all groups, no significant differences were detected between glyphosate, atrazine, and their respective mixtures (Fig. 7f-j).

### Summary of results

To provide an integrative overview of the biological responses induced by glyphosate, atrazine, and their mixtures, results obtained from the different experimental endpoints were synthesized using a descriptive comparative approach.

**Fig. 8:**
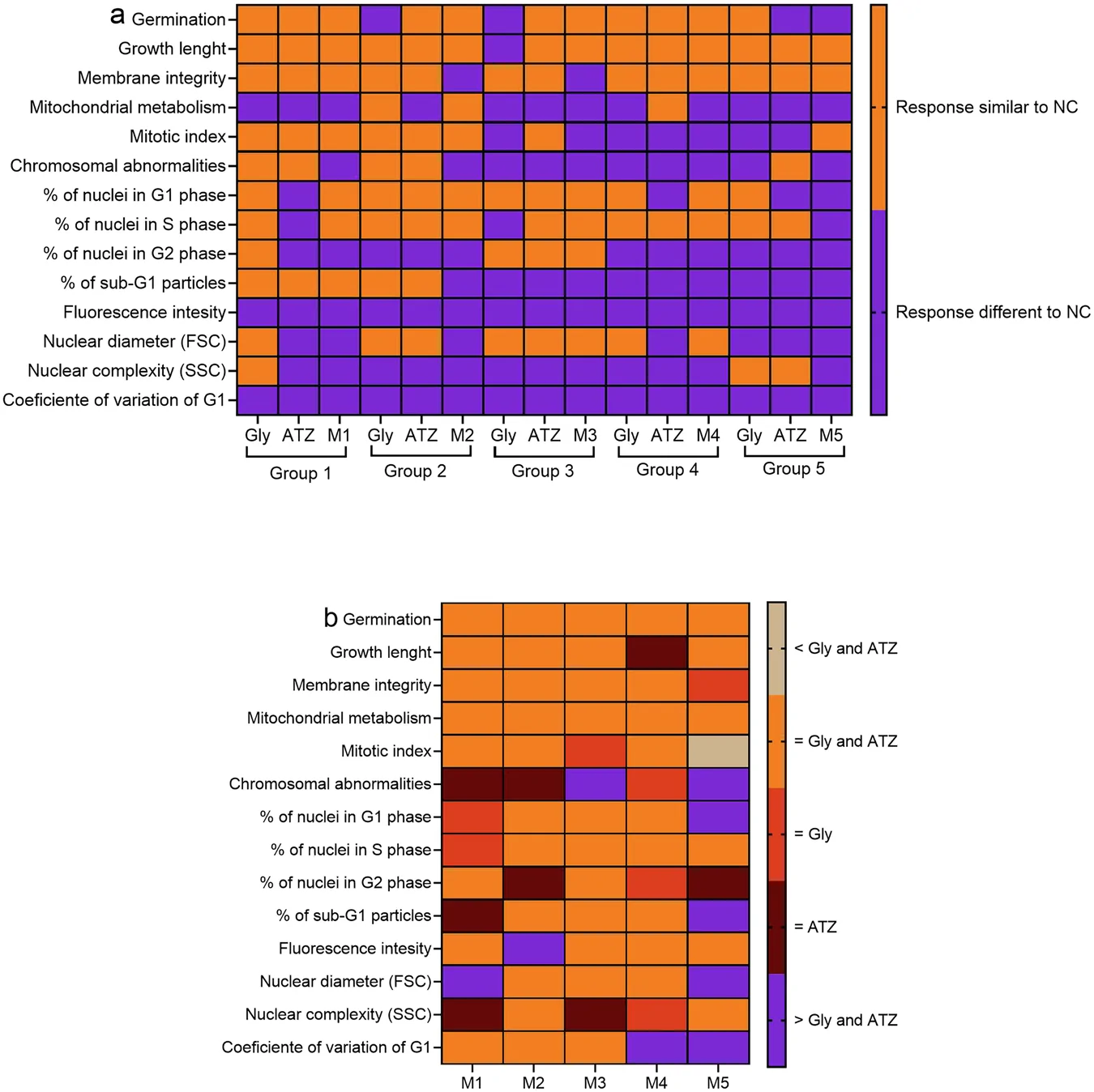
Analysis summarizing the biological responses of *A. cepa* following exposure to glyphosate (Gly), atrazine (ATZ), and their mixtures (M1-M5). **a** Orange cells represent responses statistically indistinguishable from the negative control, whereas purple cells indicate significant deviations relative to the control. **b** Comparative responses of the mixtures (M1-M5) relative to the effects of the individual herbicides. Beige cells indicate effects lower than those observed for both Gly and ATZ; orange cells indicate effects equivalent to both herbicides; red cells indicate effects comparable to either Gly or ATZ; and purple cells denote effects greater than those observed for both compounds applied separately

For this purpose, each endpoint was classified according to whether the response differed significantly from the negative control (Fig. 8a) or, in the case of mixtures, whether the response was lower than, similar to, or greater than those observed for the individual herbicides (Fig. 8b). These classifications were based exclusively on the statistical outcomes of the individual analyses described above. In addition, the proportion of endpoints showing statistically significant responses under each treatment was calculated as a descriptive metric, considering the total number of evaluated endpoints.

In Fig. 8a, each cell represents an endpoint-treatment combination that either differed significantly (purple) or did not differ significantly (orange) from the control. Overall, 113 out of the 210 evaluated combinations (53.80%) showed statistically significant differences from the control across treatments. This percentage was obtained by considering all endpoint-treatment comparisons represented in Fig. 8a, corresponding to 14 evaluated endpoints and 15 herbicide or mixture treatments. Both herbicides applied individually and their mixtures elicited significant responses in several biomarkers, although this pattern varied depending on the endpoint evaluated.

Macroscopic parameters, such as germination and root growth, as well as membrane integrity, remained largely unaffected across most treatments. In contrast, a higher frequency of significant responses was observed for parameters related to cell cycle distribution and nuclear properties, particularly at intermediary and higher concentrations (groups 3–5). Among the cytometric endpoints, fluorescence intensity and the coefficient of variation of the G₁ peak (CV-G₁) displayed the most consistent significant deviations from the control across treatments, whereas forward (FSC) and side (SSC) scatter showed a more heterogeneous pattern. The sub-G₁ fraction became more consistently significant mainly at intermediary and higher concentrations (groups 3–5). Mitochondrial metabolism and cytogenetic endpoints presented an intermediate response pattern, with significant deviations from the control occurring primarily at higher concentrations.

In Fig. 8b, colors represent the comparative response of each mixture relative to the individual herbicides. Orange tones indicate responses equivalent to both glyphosate and atrazine, red tones indicate responses comparable to one of the individual herbicides, and purple tones denote effects exceeding those observed for both compounds applied separately. Overall, most mixtures produced responses comparable to those induced by the individual herbicides. However, M5 exhibited the highest number of responses exceeding those of glyphosate and atrazine, particularly for chromosomal abnormalities, percentage of nuclei in G₁, sub-G₁ fraction, nuclear diameter (FSC), and the coefficient of variation of the G₁ peak (CV-G₁), indicating greater deviations from the responses observed under individual exposures.

## Discussion

The multiparametric approach adopted in this study demonstrated that atrazine and glyphosate, whether individually or in combination, affected multiple levels of physiological and cytogenotoxic responses in *A. cepa*.

The cytotoxic effects of the herbicides and their mixtures were evidenced by the inhibition of the mitotic index (Fig. 2d-e), a parameter directly associated with the activation of cell-cycle checkpoints and/or apoptosis induction (Bianchi et al. 2016). In general, the cell cycle is arrested at the G₁ or G₂/M phases in response to DNA damage or replication failure, allowing DNA repair enzymes to act (Bianchi et al. 2016; Moreira et al. 2021; Souza et al. 2023). This pattern was particularly observed for atrazine at 0.25 µg L⁻¹, where an increase in the frequency of G₁ cells accompanied by decreases in S and G₂/M phases was detected (Fig. 4a; f; k).

Other concentrations of atrazine, glyphosate, and their mixtures reduced both the mitotic index (Fig. 2d-e) and the frequencies of cells in G₁, S, and/or G₂/M phases (Fig. 4). This reduction may be attributed to the activation of cell death pathways, a mechanism commonly triggered when genetic damage is extensive and irreversible. As demonstrated by flow cytometry, the increase in the sub-G₁ fraction, along with the decrease in fluorescence intensity and changes in side and forward scatter parameters (SSC and FSC) (Figs. 5, 6), indicate loss of nuclear integrity, hallmarks of cell death, confirming the cytotoxic potential of the herbicides and their mixtures (Fioresi et al. 2020; Marques et al. 2025).

Cells that retain genetic damage without effective repair or apoptotic elimination may progress to mitosis, leading to an increased frequency of chromosomal abnormalities (Fioresi et al. 2020). This tendency was most evident in groups 3, 4, and 5, where micronucleus formation and chromosomal breaks predominated as the main genotoxic alterations (Table S2). The increased micronucleus frequency results from DNA strand breaks (clastogenicity) or chromosomal segregation errors (aneugenicity) (Leme and Marin-Morales, 2009).

The cytogenetic results were consistent with the increase in the G₁ peak coefficient of variation (CV) (Fig. 6f-j), a sensitive indicator of genotoxic activity in cells exposed to environmental contaminants (Bickham et al. 1992; Biradar and Rayburn, 1995; Rayburn and Wetzel, 2002; Monteiro et al. 2010). The CV reflects the homogeneity of nuclear DNA content among cells and directly represents genomic stability. Its increase suggests an uneven DNA distribution between daughter cells, consistent with the formation of micronuclei and other chromosomal aberrations (Rayburn and Wetzel, 2002; Fioresi et al. 2020; Souza et al. 2023).

Flow cytometry and cytogenetic analyses provide complementary insights into cellular responses. Flow cytometry enables the rapid analysis of thousands of nuclei, including cells in all phases of the cell cycle, thereby offering high statistical sensitivity and allowing the detection of subtle functional alterations in cell-cycle dynamics and chromatin organization. However, this technique does not permit direct visualization of mitotic structures or specific chromosomal abnormalities. Conversely, cytogenetic endpoints, such as mitotic index and chromosomal alterations, allow detailed morphological evaluation of dividing cells and precise identification of genotoxic damage, although they rely on smaller sample sizes (Marques et al. 2025). The incorporation of flow cytometry into the *A. cepa* assay broadens the methodological applicability of this classical bioindicator (Ghosh et al. 2015; Marques et al. 2025; Tavares et al. 2026).

The Evans Blue and TTC assays further confirmed that both herbicides, individually and in combination, compromised *A. cepa* root cell viability through distinct mechanisms. Among the two, the TTC assay was more sensitive, revealing that most treatments decreased mitochondrial activity (Fig. 7f-j), while only mixtures M2 and M3 disrupted plasma membrane integrity (Fig. 7b-c). These findings indicate early mitochondrial dysfunction without immediate membrane rupture. Reduced energy metabolism may impair essential processes such as cell growth, mitosis, and DNA repair (Sun et al. 2019). Such effects are compatible with apoptosis, as supported by the increase in the sub-G₁ fraction, the reductions in IFa, FSC and SSC parameters (Figs. 5, 6), and the rise in chromosomal aberrations (Fig. 2f-j).

Conversely, germination and root elongation were only slightly affected by the herbicides and mixtures (Fig. 1a-j). Regarding germination, the protective barrier provided by the seed coat likely limited direct contact between the embryo and contaminants (Rede et al. 2019). Although most treatments exhibited cytotoxic activity, as evidenced by microscopic and cytometric analyses, no significant differences were observed in root length (Fig. 1f-j). This suggests that toxic effects were restricted to the meristematic zone, without direct impact on the elongation zone, whose cells have already completed mitotic division and grow by cell expansion. The dissociation between mitotic inhibition and root elongation has been previously described (Freitas et al. 2016).

The tested concentrations and the effects reported for atrazine and glyphosate individually are consistent with previous studies. Biradar and Rayburn (1995) showed that atrazine at 0.05 μM, comparable to levels detected in groundwater in Illinois (USA), increased the CV in CHO cells. Souza et al. (2023) reported that atrazine at 1 and 2 μg L⁻¹ induced a high frequency of micronucleated cells, chromosomal bridges and losses, as well as increases in CV and sub-G₁ particles in *A. cepa*. Similarly, Felisbino et al. (2018) observed that atrazine at 1.25 μg L⁻¹ predominantly caused clastogenic damage (chromosomal bridges) in *A. cepa*.

With respect to glyphosate, Tavares et al. (2026) reported that environmentally relevant concentrations (0.1–1000 μg L⁻¹) induced cytotoxic and genotoxic effects in *A. cepa*. The consistency between these findings and the responses observed in the present study reinforces the suitability of *A. cepa* as a bioindicator of glyphosate contamination. Similar adverse effects have also been reported in aquatic organisms, supporting the broader ecological relevance of glyphosate toxicity. In this context, Rodrigues et al. (2019) identified increased DNA strand breaks after exposure to 1.7 mg L⁻¹, based on comet assay analyses. In a separate investigation, Ribas et al. (2024) demonstrated that environmentally relevant concentrations (500 μg L⁻¹) promoted cytogenetic damage, reflected by higher micronucleus incidence in circulating erythrocytes, together with oxidative imbalance and immunological suppression. These responses included reduced activities of key antioxidant enzymes in the liver, elevated lipid peroxidation, and decreased IL-1β production, with neural tissue also affected under higher exposure conditions.

Although the individual effects of glyphosate and atrazine are well known, these herbicides often co-occur in the environment and may trigger unexpected adverse responses. In the present study, most mixtures did not potentiate the effects observed for the individual compounds, suggesting independent mechanisms of action or saturation of cellular stress-response pathways. Nonetheless, this equivalence does not imply the absence of risk, since genotoxic substances lack a safe threshold of exposure (Souza et al. 2024). Therefore, even the seemingly moderate short-term effects observed here should be interpreted with caution.

Notably, mixture M5, containing concentrations comparable to those detected in agricultural surface waters, produced stronger effects than the individual compounds for several biomarkers. The responses observed for M5 may be related to the higher combined herbicide burden present in this treatment. At the highest tested concentrations, the simultaneous action of glyphosate and atrazine may increase the overall level of cellular stress, resulting in more pronounced disturbances in cell-cycle regulation, nuclear integrity, and chromosomal stability. Although the present study was not designed to formally characterize mixture interactions using specific toxicity models, the greater responses observed for M5 suggest that higher environmental contamination scenarios may represent an increased risk to non-target organisms. A similar trend was reported by Bordin et al. (2023), who observed that certain atrazine + glyphosate combinations induced higher chromosomal damage and micronucleus frequencies in *A. cepa* than single herbicides. By expanding the number of mixtures tested and including more sensitive biomarkers, the present study provides a broader and more detailed perspective on the multiparametric responses of *A. cepa* to combined exposure to glyphosate and atrazine.

## Conclusion

This study demonstrates that glyphosate and atrazine, both individually and in combination, can induce cytotoxic and genotoxic effects in *A. cepa* even at concentrations comparable to those detected in natural waters. All mixtures caused adverse effects; however, most did not potentiate the toxicity of the individual herbicides, suggesting independent or saturated mechanisms of action. The mixture containing the highest concentrations, consistent with levels reported in agricultural surface waters, exhibited stronger responses than the single compounds for some biomarkers. Endpoints derived from flow cytometric and mitochondrial assays proved to be more sensitive than traditional cytogenetic parameters, whereas germination and root growth were the least responsive. These findings highlight the value of integrating classical *A. cepa* bioassays with high-sensitivity cellular analyses to detect subtle toxic effects that precede visible morphological alterations. Overall, the results reinforce the importance of evaluating mixture toxicity under environmentally realistic conditions and support the inclusion of more sensitive biomarkers in routine ecotoxicological assessments to refine regulatory limits and strengthen water quality monitoring programs.

## Supplementary information

Supplementar Material S1

Supplementar Material S2

## Data Availability

Data will be made available on request.
