# Supplementary material for: Multilevel ecotoxicological responses of Allium cepa to atrazine and glyphosate applied individually and in combination
Source: Ecotoxicology. 2026 Sep 1;35(7):160. doi: 10.1007/s10646-026-03156-y (PMC13534172; doi:10.1007/s10646-026-03156-y)
Supplement: Supplementary file 1 [file 10646_2026_3156_MOESM1_ESM.docx]

S1. Comparison between negative and positive controls used to validate the sensitivity of the experimental system. Colchicine (0.025%) was used for germination (a), root growth (b), mitotic index (d), chromosomal abnormality (e), and flow cytometry analyses (f-l); zinc sulfate heptahydrate (6 mg L⁻¹) was used in the Evans blue assay (m); and hydrogen peroxide was used in the TTC assay (n). Statistical comparisons were performed using unpaired t- tests or Mann–Whitney tests according to data distribution (*p < 0.05; **p < 0.01; ***p < 0.001; ****p < 0.0001). Positive controls produced the expected responses for the majority of evaluated endpoints, confirming the responsiveness of the experimental system.
