# Supplementary material for: Multilevel ecotoxicological responses of Allium cepa to atrazine and glyphosate applied individually and in combination
Source: Ecotoxicology. 2026 Sep 1;35(7):160. doi: 10.1007/s10646-026-03156-y (PMC13534172; doi:10.1007/s10646-026-03156-y)
Supplement: Supplementary file 2 [file 10646_2026_3156_MOESM2_ESM.docx]

Table S2. Frequency (%) of specific chromosomal abnormalities observed in meristematic cells of *A. cepa* after exposure to glyphosate and atrazine, and their mixtures (M1 to M5)

| Treatments | | Specific chromosomal abnormalities (%) | | | | | | | | |
| --- | --- | --- | --- | --- | --- | --- | --- | --- | --- | --- |
|  |  | Chromosomal adherence | Chromosomal break | Chromosomal Bridge | Nuclear Bud | C-Metaphase | Chromosomal Loss | Anaphase  Multipolar | Micronucleus | Polyploidy |
| NC |  | 0 ± 0 | 0.07 ± 0.08 | 0.07 ± 0.05 | 0 ± 0 | 0.03 ± 0.05 | 0 ± 0 | 0 ± 0 | 0.32 ± 0.09 | 0 ± 0 |
| GLY μg L⁻¹ | 62.5 | 0.03 ± 0.05 | 0.17 ± 0.10 | 0.02 ± 0.04 | 0 ± 0 | 0 ± 0 | 0 ± 0 | 0 ± 0 | 0.35 ± 0.31 | 0 ± 0 |
|  | 125 | 0 ± 0 | 0.02 ± 0.04 | 0.03 ± 0.05 | 0.02 ± 0.04 | 0.06 ± 0.05 | 0 ± 0 | 0 ± 0 | 0.60 ± 0.81 | 0 ± 0 |
|  | 250 | 0.03 ± 0.05 | 0.16 ± 0.14 | 0.03 ± 0.05 | 0 ± 0 | 0.17 ± 0.14 | 0.21 ± 0.33 | 0 ± 0 | 1.0 ± 0.26 | 0 ± 0 |
|  | 500 | 0.11 ± 0.13 | 0.65 ± 0.50 | 0 ± 0 | 0.10 ± 0.09 | 0.02 ± 0.04 | 0.13 ± 0.19 | 0 ± 0 | 1.16 ± 0.20 | 0 ± 0 |
|  | 1000 | 0.17 ± 0.40 | 1.03 ± 0.45 | 0.06 ± 0.12 | 0.16 ± 0.12 | 0.02 ± 0.04 | 0.28 ± 0.21 | 0 ± 0 | 2.36 ± 0.59 | 0 ± 0 |
| ATZ μg L⁻¹ | 0.25 | 0 ± 0 | 0.20 ± 0.09 | 0 ± 0 | 0 ± 0 | 0.03 ± 0.08 | 0 ± 0 | 0.53 ± 0.23 | 0 ± 0 | 0 ± 0 |
|  | 0.5 | 0.36 ± 0.80 | 0.16 ± 0.22 | 0.01 ± 0.04 | 0 ± 0 | 0.05 ± 0.08 | 0.05 ± 0.08 | 0 ± 0 | 0.35 ± 0.13 | 0 ± 0 |
|  | 1 | 0 ± 0 | 0.28 ± 0.17 | 0.05 ± 0.05 | 0.08 ± 0.07 | 0.05 ± 0.12 | 0.03 ± 0.05 | 0.02 ± 0.04 | 0.95 ± 0.51 | 0 ± 0 |
|  | 2 | 0 ± 0 | 0.31 ± 0.15 | 0.02 ± 0.04 | 0.1 ± 0.1 | 0.08± 0.09 | 0.27 ± 0.38 | 0.02 ± 0.04 | 1.0 ± 0.26 | 0 ± 0 |
|  | 4 | 0.03 ± 0.05 | 0.22 ± 0.11 | 0.06 ± 0.08 | 0 ± 0 | 0.18 ± 0.21 | 0.16 ± 0.22 | 0 ± 0 | 0.9 ± 0.17 | 0 ± 0 |
| Mixtures | M1 | 0.10 ± 0.08 | 0.13 ± 0.15 | 0.05 ± 0.05 | 0.02 ± 0.04 | 0.18 ± 0.15 | 0.15 ± 0.08 | 0.02 ± 0.04 | 1.40 ± 0.60 | 0.02 ± 0.04 |
|  | M2 | 0.13 ± 0.12 | 0.43 ± 0.25 | 0.10 ± 0.08 | 0.08 ± 0.07 | 0.08 ± 0.16 | 0.10 ± 0.10 | 0.02 ± 0.04 | 2.20 ± 0.97 | 0.02 ± 0.04 |
|  | M3 | 0.18 ± 0.19 | 1.33 ± 0.20 | 0.02 ± 0.04 | 0.06 ± 0.08 | 0.21 ± 0.24 | 0.25 ± 0.15 | 0.02 ± 0.04 | 1.46 ± 0.46 | 0.03 ± 0.05 |
|  | M4 | 0.13 ± 0.12 | 0.78 ± 0.37 | 0.03 ± 0.05 | 0.08 ± 0.07 | 0.26 ± 0.37 | 0.10 ± 0.10 | 0 ± 0 | 1.5 ± 0.5 | 0 ± 0 |
|  | M5 | 0.36 ± 0.26 | 0.65 ± 0.68 | 0.10 ± 0.08 | 0.11 ± 0.14 | 0.70 ± 0.49 | 0.25 ± 0.13 | 0.13 ± 0.12 | 4.48 ± 1.4 | 0.03 ± 0.08 |

Values represent the frequency (%) of each chromosomal abnormalities type calculated relative to the total number of meristematic cells scored per slide (mean ± SD).
